# Expanding the toolbox of ADHD genetics. How can we make sense of parent of origin effects in ADHD and related behavioral phenotypes?

**DOI:** 10.1186/s12993-015-0078-4

**Published:** 2015-10-16

**Authors:** Tetyana Zayats, Stefan Johansson, Jan Haavik

**Affiliations:** Department of Biomedicine, K.G. Jebsen Centre for Neuropsychiatric Disorders, University of Bergen, Bergen, Norway; Department of Clinical Science, K.G. Jebsen Centre for Neuropsychiatric Disorders, University of Bergen, Bergen, Norway; Center for Medical Genetics and Molecular Medicine, Haukeland University Hospital, Bergen, Norway; Division of Psychiatry, Haukeland University Hospital, Bergen, Norway

**Keywords:** ADHD, Parent-of-origin effects, Imprinting, Maternal effects, Mitochondrial DNA, Sex chromosomes, Epigenetics

## Abstract

Genome-wide association (GWA) studies have shown that many different genetic variants cumulatively contribute to the risk of psychiatric disorders. It has also been demonstrated that various parent-of-origin effects (POE) may differentially influence the risk of these disorders. Together, these observations have provided important new possibilities to uncover the genetic underpinnings of such complex phenotypes. As POE so far have received little attention in neuropsychiatric disorders, there is still much progress to be made. Here, we mainly focus on the new and emerging role of POE in attention-deficit hyperactivity disorder (ADHD). We review the current evidence that POE play an imperative role in vulnerability to ADHD and related disorders. We also discuss how POE can be assessed using statistical genetics tools, expanding the resources of modern psychiatric genetics. We propose that better comprehension and inspection of POE may offer new insight into the molecular basis of ADHD and related phenotypes, as well as the potential for preventive and therapeutic interventions.

## Background

Genome-wide association (GWA) studies have demonstrated that many different genetic variants cumulatively contribute to the risk of psychiatric disorders. The GWA approach has been particularly successful in schizophrenia, where over 108 susceptibility loci have been identified [1]. Despite this recent progress, little is yet known of the precise genes and molecular mechanisms involved, such as epigenetic risk factors transmitted across generations. Moreover, the molecular genetics of other, more prevalent, psychiatric disorders are even less established. This includes attention-deficit hyperactivity disorder (ADHD), which is one of the most common and most heritable childhood-onset mental disorders [2, 3].

Even with considerable research efforts being directed towards understanding the etiology of ADHD and clearly identifying a wide range of risk factors, genome-wide studies have, so far, failed to identify robustly associated genes. The majority of candidate genes studied to date confer only a small risk for ADHD, often refer to co-occurring conditions and they lack consistent replication [4]. Apart from the fact that ADHD is a multifactorial disorder and previous GWA studies have been underpowered, this may also be due to a number of other factors, such as phenotypic heterogeneity, rare and unexplored variants, epistasis, gene × environment interaction as well as the presence of parent-of-origin effects (POE). Compared to the efforts of increasing sample size, evaluating rare variants and gene x environment interaction studies, the elucidation of POE has been lagging behind, although, with large enough sample, POE may be detected indirectly through offspring’s own genotype effect [5, 6]. Here, we review the main concepts of POE and how this knowledge can be applied to studies of neuropsychiatric disorders, such as ADHD.

## Parent-of-origin effects (POE)

**Fig. 1:**
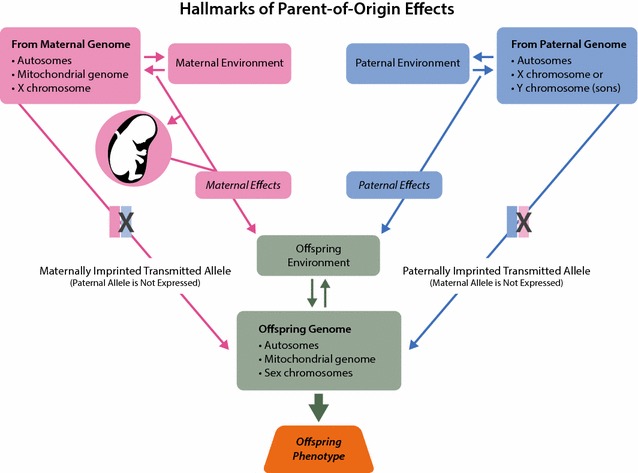
Hallmarks of parent-of-origin effects. The main mechanisms of parent-of-origin effects are depicted in the form of the genetic and environmental components that are affecting the development of a phenotype in an offspring

POE is a collective term referring to situations in which mothers and fathers do not contribute equally to the development of a phenotype in their offspring [7]. There are several known genetic mechanisms that could account for differential influence of one parent or the other: (1) genomic imprinting, (2) effects of the maternal genome on intrauterine environment and fetus (maternal effects) and (3) mitochondrial genome and sex chromosomes. In addition, many of these effects may have environmental interaction and epistatic components [8] (Fig. 1).

### Genomic imprinting

Genomic imprinting is the occurrence of monoallelic expression of only one of the two alleles, dictated by parental origin [9]. Such differential expression of paternal versus maternal alleles is mediated by epigenetics [10], defined as heritable alterations in gene expression caused by mechanisms other than changes in DNA sequence [11]. Main epigenetic components known to be involved in imprinting are modifications in chromatin and methylation of DNA itself [12].

Imprinting has already been implicated in psychiatric health [13, 14], with documented evidence in schizophrenia [15, 16], autism [17, 18] and bipolar disorder [19, 20]. The variability in ADHD phenotype was shown to differ depending on parental ADHD history [21] and some rare genetic syndromes with ADHD symptomatology exhibit clinical evidence of imprinting [22]. However, our knowledge about imprinting in ADHD is still rudimentary, with small studies and conflicting results dominating the field. Some ADHD candidate genes have been shown to have a strongly biased pattern of parental transmission in brain tissue [23, 24]. Early reports of asymmetric parental transmission to ADHD affected offspring have implicated several genes, including *SNAP25* [25–27], *HTR1B* [28–30], *SLC6A4* [31, 32], *BDNF* [33], *DDC* [34], *GNAL* [35], *TPH2*, *DRD4*, *DRD5* [31] and *SLC6A3* [31, 36]. These candidate gene findings have been reported in the early 2000s, with the last one published in 2010. As fundamental studies in neurobiology revealed that neuropsychiatric conditions are most likely to be polygenic, combined with overall low reproducibility of candidate gene studies, hypothesis-free genome-wide association (GWA) studies have taken over the field of psychiatric genetics in the past decade. In addition to allowing the interrogation of the entire genome, GWA studies may also benefit from a case/control design, where association signal is derived from unrelated individuals, without the need for parental genotypes. Such approach is particularly appealing in studies of late-onset neuropsychiatric disorders, where parent samples may not be obtained. The concept of genome-wide interrogation, nonetheless, can also be applied outside genetic association and case/control setup. POE, for example, can also be examined throughout the entire genome in trio data. However, we only know of a single genome-wide examination of imprinting in ADHD reported in 2012, with suggested parent-of-origin effects (not reaching genome-wide significance) in *FAS* and *PDLIM1* genes [37]. In comparison, over 10 neuropsychiatric GWA studies have been published since 2008 (Catalogue of Genome Wide Association Studies, accessed in august 2015). Such gap in the amount of GWA studies versus genome-wide POE studies may be due to a number of reasons, as, for example, difficulties of collecting large number of trios and/or lack of guidance in the correct use of statistical models to test POE under various scenarios [38].

DNA methylation patterns have also been studied in ADHD, mainly in previously nominated candidate genes. A recent study of dopamine transporter gene *SLC6A3*, that has been suggested to be imprinted, highlights several methylation-prone features of its sequence, including over 90 satellite repeats in its body and high cytosine/guanine density in its promoter region [39]. A longitudinal examination of DNA methylation across genes previously associated with ADHD suggests that methylation differences in those genes are already apparent in early childhood even among identical twins, and that individual changes in methylation are not stable over time [40]. Nonetheless, to which extent methylation patterns in ADHD reflect genomic imprinting remains to be determined.

### Maternal effects

Parent-of-origin effects are often interpreted as evidence of imprinting. However, there are additional mechanisms that can lead to differential impact of parents (especially mothers) on their offspring. At birth, the phenotype of a new-born may have already been strongly affected by the intrauterine milieu [41]. The intrauterine environment is partly shaped by the maternal genome. In this respect, the maternal genome, regardless of which alleles are directly passed on to the offspring, can exert specific influence on offspring phenotypes. Parental genetic effects may occur when an allele expressed in only mothers or fathers has a causal influence on the phenotype of an offspring [42]. Such effects have an environmental interaction or epistatic component [8].

Maternal genetic variants with a strong impact on fetal development have been studied in clinical genetics, where they have been termed “teratogenic alleles”. By similar mechanisms, the risk of psychiatric disorders in the offspring may also be influenced by maternal genes [43]. For instance, genes related to the immune system could both be considered as risk genes themselves and as mediators of POE. In addition, neonatal development can be modified by maternal care and show a POE [44]. It is also noteworthy, that while we here focus on maternal effects, paternal effects are also possible, although these effects have been less studied.

However, in practice it is difficult to sort out exactly how maternally or paternally inherited alleles would impact the developing fetus’ response to intrauterine environment (epistasis and gene × environment interaction). Maternally derived hormonal factors are known to influence prenatal growth and development. For instance, animal studies have shown that maternally derived serotonin can influence embryonic development. In ADHD, it was shown that maternal *TPH1* mutations resulting in impaired maternal serotonin production may have long-term consequences for brain-development and increase the risk of ADHD-related symptoms in their offspring [45]. Similarly, human prescription registry data have indicated that prenatal exposure to serotonin reuptake inhibitors has a lasting effect on offspring behavior [46]. A similar maternal effect was also shown in a mouse model of anxiety, where the offspring of mothers heterozygote for a knockout mutation of the serotonin A1 receptor exhibited anxiety-like phenotype, even without inheriting the mutation themselves [47].

### Mitochondrial genome and sex chromosomes

Mitochondrial DNA and the non-pseudoautosomal region of Y chromosome (which is only transmitted from father to son) are the two particular cases of POE. Classical mitochondrial diseases are characterized by maternal inheritance as well as muscular and neurological dysfunction. Similarly, polymorphisms in mitochondrial DNA have been also associated with neuropsychiatric conditions, such as schizophrenia, bipolar disorder, major depressive disorder and autism [48–50]. The mitochondrial genome is considered to be exclusively maternally inherited [51], although there is also an example of paternal inheritance of a mitochondrial disease in humans [52]. Several studies indicate that mitochondrial DNA contributes to synaptic transmission [53] and calcium signaling [54], important brain functions that also have been implicated in neuropsychiatric disorders [55].

Apart from being maternally inherited, mitochondrial DNA may also exert its parent-of-origin effect through modification of penetrance of nuclear genome. Such a phenomenon has been described in a case of a familial neuropathy caused by mutations in the autosomal *TTR* gene [56, 57].

The non-pseudoautosomal region of the Y chromosome is exclusively transmitted from father to son [8]. Animal studies show that mice and rat lines that differ only in that particular Y chromosomal region reveal altered brain morphology and behavior [58, 59]. Y-chromosome genes may contribute to our understanding of neurodevelopmental disorders exhibiting sexual dimorphisms, such as autism, ADHD, obsessive–compulsive disorder and major depressive disorder. It has been shown, for example, that the Y-encoded SRY (sex-determining region Y) transcription factor modulates MAOA (monoamine oxidase A) activity in human male neuroblastoma cells [60]. *MAOA* is encoded on the X chromosome, plays vital role in the metabolism of neurotransmitters and its dysfunctions have been implicated in several neuropsychiatric conditions, including ADHD [61]. Thus, Y-chromosome located genes may be able to regulate X-encoded genes, providing molecular mechanism for sexual dimorphisms in neuropsychiatric disorders.

Behavioral changes linked to sex chromosomes can also be observed in Turner’s and Klinefelter’s syndromes, with abnormal behavioral/cognitive phenotypes depending on parental origin of X chromosome. Different levels of autistic symptoms have been reported between Klinefelter’s patients with additional maternal or paternal X chromosome [62], while paternally derived X chromosome in Turner’s syndrome has been associated with improvement in social cognition [63].

## Statistical methods to detect POE in humans: what is missing in GWA studies

GWA studies have contributed to a substantial advancement of human genetic knowledge during the past decade, but these data can so far only explain a fraction of the estimated heritability of complex disorders. In addition, associations detected in case/control design may originate from genetic mechanisms other than those functioning in cases, but rather from their confounders, such as maternal genotype effects, imprinting and/or maternal-fetal interactions [5, 6]. Importantly, the panel of statistical tools used in psychiatric genetics should also include models allowing for the presence of POE.

### Methods utilizing trio data

One aspect of POE, the examination of which has been attempted the most, is imprinting, or non-equivalence, of parental alleles. To date, all studies of POE in ADHD have applied transmission disequilibrium test (TDT) based approaches to identify biased transmission of parental alleles [31, 33]. In short, a 2 × 2 table containing transmitted/non-transmitted allele counts according to the maternal or paternal origin of the alleles is constructed and Fisher’s exact test or Chi square test is applied to compare those. A similar approach is also implemented in the widely used PLINK’s parent-of-origin test [64]. Although the TDT has been proven to be a useful tool in POE analyses [65], this method has some weaknesses: (1) *null hypothesis examined* the hypothesis tested by TDT itself is that of random meiotic selection of a parental allele for transmission to an offspring, but in POE it is the equality of paternal and maternal transmissions; (2) *non*-*independence of observations* the TDT statistics assumes that observed transmissions are independent (under Mendel’s second law), meaning the TDT-based parental transmission comparison may not be valid because of the statistical dependence between maternal and paternal transmissions in triads with two heterozygous parents or because of the possible prenatal maternal effect occurring together with genetic association in triads with homozygote parents [38, 66]; (3) *bias due to missing data* POE detection by TDT-based approach may introduce likely bias when incomplete trios are utilized (for example, a trio with genotyped mother only will contribute to overall association test and to the test of maternal transmissions, but not to that of paternal transmissions) and (4) *confounding effects* TDT-based method to study POE is sensitive to a possible confounding impact of maternal effects on non-equivalence of parental transmissions [38, 67], as the two cannot be clearly distinguished with this test. Such methodology together with insufficient correction for multiple testing and rather modest sample sizes makes it difficult to draw firm conclusions. In fact, the outcomes of some of these studies on ADHD have either been subject of much controversy [68, 69] or have failed to replicate [69–72].

One method to account for both maternal effects and non-equivalence of parental alleles is the application of a log-linear modeling in trio data, that can be implemented in a number of softwares (e.g. EMIM/PREMIM, HAPLIN) [5, 38, 73–75]. A likelihood-based approach provides a valid substitute to TDT-based method by performing stratification on both the parental mating type and the inherited number of copies of the allele under study [66], allowing for reliable testing of both imprinting and maternal effects. In addition, the likelihood ratio test (LRT) of the log-linear modeling performs better than TDT under dominant or recessive models [73, 76]. LRT also allows for possible prenatal maternal genetic effects [73, 77] and incomplete triads [78]. It is not only robust in detection of POE, but is also able to provide insight into distinguishable imprinting and maternal effects. Furthermore, incorporation of parental origin into association studies may lead to detection of overall genetic effect of an allele (POE and association), possibly incrementing the power of a GWA study and, thus, reducing the sample size needed to observe an association signal, although the required sample size, most likely, still needs to be larger than the ones examined so far.

To date, log-linear modeling has not been applied to analyze POE in ADHD.

### Methods utilizing unrelated individuals

Apart from the family-based TDT approach, the parental origin of a genetic polymorphism may also be assessed in a sample of unrelated individuals by comparing phenotypic variance of a quantitative trait in a heterozygous genotype group to that of a homozygous group [79]. This test assumes that a heterozygous group would exhibit increased phenotypic variability compared to a homozygous group, because it consists of two subgroups of reciprocal heterozygotes (paternal reference allele/maternal alternative allele and visa versa) each with different phenotypic means. While this method has the benefit of analyzing POE without trios, opening new potential of detecting POE in GWA data, it can lead to spurious conclusions when parental genetic effects are present (e.g., maternal effect).

Another approach that can utilize unrelated individuals to examine POE is Mendelian randomization (MR), an alternative method to investigate the causal nature of early life events (e.g., intrauterine fetal development) on disorders developed later in life. MR applies genetic variants to infer about the effect of a non-genetic risk factor (e.g., alcohol consumption during pregnancy) on a disease outcome (e.g., ADHD). Genetic variants employed in MR must be associated with the disorder in question, serving as a proxy for it. The alleles of such variants can then be used to divide a population into groups that differ systematically only in the presence of the examined disorder. Since the association between these variants and a disorder is not generally confounded by behavioral or environmental exposures, such design provides a causal estimate that is free from confounding, analogous to randomized controlled trials [80]. MR was implemented, for example, to elucidate the detrimental role of intrauterine alcohol exposure on cognitive performance later in life [81]. Nonetheless, while MR may be a useful tool to examine POE, its underlying main assumption of a strong association between the utilized genetic variants and the examined disorder is challenging to meet for ADHD, as, to date, no genome-wide association was reported for this condition. If the association between genetic polymorphisms and the phenotype is not substantial, the causal estimate will be biased in the direction of weak association, giving rise to erroneous conclusion of causality [80].

## Review

The role of POE in the development of ADHD is alluring, and we have only just begun to explore these phenomena. The phenotypes, that have already been shown to be influenced by POE, suggest that these effects may have important impact on the development of neuropsychiatric conditions, including ADHD. Nonetheless, the exploration of POE in ADHD has been limited to mostly candidate gene studies, with no consistent findings thus far. Genome-wide analyses of POE mechanisms are also lacking in ADHD, as there has only been published a single genome-wide parent-of-origin effect study on ADHD, performed on a relatively under-powered sample of 846 trios [37].

As the genetic association is largely conceptualized by loci with two equivalent alleles, most GWA studies of ADHD and related neuropsychiatric phenotypes have not implemented POE. Instead, genetic studies of POE in ADHD have been utilizing family-based TDT-like statistics to examine mostly just candidate genes. However, such approach may result in outcomes that are difficult to interpret as this test cannot provide clear distinction between maternal effects and non-equivalence of parental transmissions (imprinting). This may affect the formation of new hypotheses about molecular mechanisms of ADHD and the design of follow up functional studies. A presumption of imprinting would prompt the examination of epigenetic mechanisms in affected offsprings, while that of maternal effect would drive the evaluation of maternal genome and environment (including intrauterine environment). Thus, psychiatric genetics may benefit from the implementation of alternative statistical methods (e.g. LRT) to detect POE as the number, the impact and the function of genes with parent-of-origin effects in ADHD remains an important open question.

While improving statistical methods to examine POE in neuropsychiatric conditions will advance our odds of uncovering such effects, it is worth mentioning that sample size is a critical factor as well. Collecting trio data is challenging and, in many instances, recruited trios are incomplete, with missing parental genotypes adding to the difficulties of achieving adequate sample sizes. This is of particular concern in recruitment of trios with late-onset neuropsychiatric conditions (e.g., major depressive disorder), where parental genotypes may not be attainable. Recently, the formation of the international psychiatric genetics consortium (PGC) provided a platform for a much needed boost in assembling large enough samples, which led to identification of a number of genome-wide significant association loci for neuropsychiatric disorders [1, 82]. Nonetheless, the collection of trios is still not as efficient as that of unrelated individuals: compared to over 17.000 cases and 94.000 controls, only 2.064 trios were collected for ADHD to date. Apart from direct recruitment of trios, one way to circumvent the obstacle of small sample size is to turn to statistical tools to utilize incomplete trios (mother/child or father/child duos) by carrying out analyses at multiple variants simultaneously (haplotype analyses) and/or by relying on the expectation–maximization algorithm to estimate the missing genotypes [5, 74]. Greater power may also be achieved by analyzing trios together with samples of unrelated controls, which can provide a measure of reference allele frequency and take advantage of already collected large-scale samples, such as those of PGC [5]. Regardless of these techniques, however, the majority of methods to detect POE generally assume that both parents are available for at least a subset of affected cases [5]. Thus, unraveling practical issues of accumulating large number of trios with an affected offspring as well as advancing our statistical methods will aid POE analyses in complex disorders.

## Conclusions

Research into POE of neuropsychiatric disorders is still in its methodological infancy and has not yet produced genome-wide significant and replicable results. The development and application of modern statistical approaches to examine the genetics of ADHD beyond simple association can yield novel insights into the genetic architecture of this disorder. Considering POE as a source of phenotypic variation can reveal a “hidden” heritable component that remains unrecognized in traditional association studies of complex traits. In addition, POE could add to the incomplete monozygotic twin concordance often observed for ADHD behavior [83], the phenomenon of the sexually dimorphic (male) vulnerability to ADHD and offer a ramification to traditional approaches of disease gene identification, explaining why it is so difficult to pinpoint specific casual genes in apparently highly heritable disorders like ADHD.

In conclusion, better comprehension and inspection of POE may offer new possibilities of gaining insight into the molecular basis of ADHD and related phenotypes, as well as the potential for preventive and therapeutic interventions.

## Abbreviations

GWA: Genome-wide association
ADHD: Attention deficit hyperactivity disorder
POE: Parent-of-origin effects
TDT: Transmission disequilibrium test
LRT: Likelihood ratio test

## References

[1] Schizophrenia Working Group of the Psychiatric Genomics C. (2014). Biological insights from 108 schizophrenia-associated genetic loci. Nature.

[2] Faraone SV (2005). Molecular genetics of attention-deficit/hyperactivity disorder. Biol Psych.

[3] Polanczyk G (2007). The worldwide prevalence of ADHD: a systematic review and metaregression analysis. Am J Psych.

[4] Kebir O (2009). Candidate genes and neuropsychological phenotypes in children with ADHD: review of association studies. J Psych Neurosci.

[5] Ainsworth HF (2011). Investigatio of maternal effects, maternal-fetal interactions and parent-of-origin effects (imprinting), using mothers and their offspring. Genet Epidemiol.

[6] Buyske S (2008). Maternal genotype effects can alias case genotype effects in case–controls studies. Eur J Hum Genet.

[7] Curley JP, Mashoodh R (2010). Parent-of-origin and trans-generational germline influences on behavioral development: the interacting roles of mothers, fathers, and grandparents. Dev Psychobiol.

[8] Guilmatre A, Sharp AJ (2012). Parent of origin effects. Clin Genet.

[9] Reik W, Walter J (2001). Genomic imprinting: parental influence on the genome. Nat Rev Genet.

[10] de la Casa-Esperon E, Sapienza C (2003). Natural selection and the evolution of genome imprinting. Annu Rev Genet.

[11] Egger G (2004). Epigenetics in human disease and prospects for epigenetic therapy. Nature.

[12] Jaenisch R, Bird A (2003). Epigenetic regulation of gene expression: how the genome integrates intrinsic and environmental signals. Nat Genet.

[13] Badcock C, Crespi B (2008). Battle of the sexes may set the brain. Nature.

[14] Isles AR, Wilkinson LS (2000). Imprinted genes, cognition and behaviour. Trend Cogn Sci.

[15] Pun FW (2011). Imprinting in the schizophrenia candidate gene GABRB2 encoding GABA(A) receptor beta(2) subunit. Mol Psych.

[16] Ludwig KU (2009). Supporting evidence for LRRTM1 imprinting effects in schizophrenia. Mol Psych.

[17] Skuse DH (2000). Imprinting, the X-chromosome, and the male brain: explaining sex differences in the liability to autism. Pediatr Res.

[18] Badcock C, Crespi B (2006). Imbalanced genomic imprinting in brain development: an evolutionary basis for the aetiology of autism. J Evol Biol.

[19] McMahon FJ (1995). Patterns of maternal transmission in bipolar affective disorder. Am J Hum Genet.

[20] Borglum AD (2003). Possible parent-of-origin effect of Dopa decarboxylase in susceptibility to bipolar affective disorder. Am J Med Genet B Neuropsychiatr Genet.

[21] Goos LM, Ezzatian P, Schachar R (2007). Parent-of-origin effects in attention-deficit hyperactivity disorder. Psych Res.

[22] Lichter DG, Jackson LA, Schachter M (1995). Clinical evidence of genomic imprinting in Tourette’s syndrome. Neurology.

[23] Crowley JJ (2015). Analyses of allele-specific gene expression in highly divergent mouse crosses identifies pervasive allelic imbalance. Nat Genet.

[24] Jacobsen KK et al. Epistatic and gene wide effects in YWHA and aromatic amino hydroxylase genes across ADHD and other common neuropsychiatric disorders: association with YWHAE. Am J Med Genet B Neuropsychiatr Genet. 2015. 10.1002/ajmg.b.32339 PMC5034749 26172220

[25] Brophy K (2002). Synaptosomal-associated protein 25 (SNAP-25) and attention deficit hyperactivity disorder (ADHD): evidence of linkage and association in the Irish population. Mol Psych.

[26] Kustanovich V (2003). Biased paternal transmission of SNAP-25 risk alleles in attention-deficit hyperactivity disorder. Mol Psych.

[27] Mill J (2004). Haplotype analysis of SNAP-25 suggests a role in the aetiology of ADHD. Mol Psych.

[28] Hawi Z (2002). Serotonergic system and attention deficit hyperactivity disorder (ADHD): a potential susceptibility locus at the 5-HT(1B) receptor gene in 273 nuclear families from a multi-centre sample. Mol Psych.

[29] Quist JF (2003). The serotonin 5-HT1B receptor gene and attention deficit hyperactivity disorder. Mol Psych.

[30] Smoller JW (2006). Association between the 5HT1B receptor gene (HTR1B) and the inattentive subtype of ADHD. Biol Psych.

[31] Hawi Z (2005). Preferential transmission of paternal alleles at risk genes in attention-deficit/hyperactivity disorder. Am J Hum Genet.

[32] Banerjee E (2006). A family-based study of Indian subjects from Kolkata reveals allelic association of the serotonin transporter intron-2 (STin2) polymorphism and attention-deficit-hyperactivity disorder (ADHD). Am J Med Genet B Neuropsychiatr Genet.

[33] Kent L (2005). Association of the paternally transmitted copy of common Valine allele of the Val66Met polymorphism of the brain-derived neurotrophic factor (BDNF) gene with susceptibility to ADHD. Mol Psych.

[34] Hawi Z (2001). Dopa decarboxylase gene polymorphisms and attention deficit hyperactivity disorder (ADHD): no evidence for association in the Irish population. Mol Psych.

[35] Laurin N (2008). Investigation of the G protein subunit Galphaolf gene (GNAL) in attention deficit/hyperactivity disorder. J Psychiatr Res.

[36] Hawi Z (2010). ADHD and DAT1: further evidence of paternal over-transmission of risk alleles and haplotype. Am J Med Genet B Neuropsychiatr Genet.

[37] Wang KS (2012). Parent-of-origin effects of FAS and PDLIM1 in attention-deficit/hyperactivity disorder. J Psych Neurosci.

[38] Connolly S, Heron E (2014). Review of statistical methodologies for the detection of parent-of-origin effects in family trio genome-wide association data with binary disease traits. Brief Bioinform.

[39] Shumay E, Fowler JS, Volkow ND (2010). Genomic features of the human dopamine transporter gene and its potential epigenetic states: implications for phenotypic diversity. PLoS One.

[40] Wong CC (2010). A longitudinal study of epigenetic variation in twins. Epigenetics.

[41] Rampersaud E (2008). Investigating parent of origin effects in studies of type 2 diabetes and obesity. Curr Diabet Rev.

[42] Wolf JB, Wade MJ (2009). What are maternal effects (and what are they not)?. Philos Trans R Soc Lond B Biol Sci.

[43] Haavik J (2011). Maternal genotypes as predictors of offspring mental health: the next frontier of genomic medicine?. Futur Neurol.

[44] Hager R, Cheverud JM, Wolf JB (2009). Change in maternal environment induced by cross-fostering alters genetic and epigenetic effects on complex traits in mice. Proc Biol Sci.

[45] Halmoy A (2010). Attention-deficit/hyperactivity disorder symptoms in offspring of mothers with impaired serotonin production. Arch Gen Psych.

[46] Moses-Kolko EL (2005). Neonatal signs after late in utero exposure to serotonin reuptake inhibitors: literature review and implications for clinical applications. JAMA.

[47] Gleason G (2010). The serotonin1A receptor gene as a genetic and prenatal maternal environmental factor in anxiety. Proc Natl Acad Sci.

[48] Martorell L (2006). New variants in the mitochondrial genomes of schizophrenic patients. Eur J Hum Genet.

[49] Rollins B (2009). Mitochondrial variants in schizophrenia, bipolar disorder, and major depressive disorder. PLoS One.

[50] Weissman JR (2008). Mitochondrial disease in autism spectrum disorder patients: a cohort analysis. PLoS One.

[51] Giles RE (1980). Maternal inheritance of human mitochondrial DNA. Proc Natl Acad Sci.

[52] Schwartz M, Vissing J (2002). Paternal inheritance of mitochondrial DNA. N Engl J Med.

[53] Billups B, Forsythe ID (2002). Presynaptic mitochondrial calcium sequestration influences transmission at mammalian central synapses. J Neurosci.

[54] Kubota M (2006). Abnormal Ca2+ dynamics in transgenic mice with neuron-specific mitochondrial DNA defects. J Neurosci.

[55] Kiser DP, Rivero O, Lesch KP (2015). Annual research review: the (epi)genetics of neurodevelopmental disorders in the era of whole-genome sequencing–unveiling the dark matter. J Child Psychol Psych.

[56] Olsson M (2009). Mitochondrial haplogroup is associated with the phenotype of familial amyloidosis with polyneuropathy in Swedish and French patients. Clin Genet.

[57] Bonaiti B (2009). Parent-of-origin effect in transthyretin related amyloid polyneuropathy. Amyloid.

[58] Sluyter F (1996). Aggression in wild house mice: current state of affairs. Behav Genet.

[59] Guillot PV (1996). Hippocampal morphology in the inbred mouse strains NZB and CBA/H and their reciprocal congenics for the nonpseudoautosomal region of the Y chromosome. Behav Genet.

[60] Wu JB (2009). Regulation of monoamine oxidase A by the SRY gene on the Y chromosome. FASEB J.

[61] Gizer IR, Ficks C, Waldman ID (2009). Candidate gene studies of ADHD: a meta-analytic review. Hum Genet.

[62] Bruining H (2010). The parent-of-origin of the extra X chromosome may differentially affect psychopathology in Klinefelter syndrome. Biol Psych.

[63] Skuse DH (1997). Evidence from Turner’s syndrome of an imprinted X-linked locus affecting cognitive function. Nature.

[64] Purcell S (2007). PLINK: a tool set for whole-genome association and population-based linkage analyses. Am J Hum Genet.

[65] Wallace C (2010). The imprinted DLK1-MEG3 gene region on chromosome 14q32.2 alters susceptibility to type 1 diabetes. Nat Genet.

[66] Weinberg CR (1999). Methods for detection of parent-of-origin effects in genetic studies of case-parents triads. Am J Hum Genet.

[67] Hager R, Cheverud JM, Wolf JB (2008). Maternal effects as the cause of parent-of-origin effects that mimic genomic imprinting. Genetics.

[68] Joober R, Sengupta S (2006). Parent-of-origin effect and risk for attention-deficit/hyperactivity disorder: balancing the evidence against bias and chance findings. Am J Hum Genet.

[69] Laurin N (2007). No preferential transmission of paternal alleles at risk genes in attention-deficit hyperactivity disorder. Mol Psych.

[70] Anney RJ (2008). Parent of origin effects in attention/deficit hyperactivity disorder (ADHD): analysis of data from the international multicenter ADHD genetics (IMAGE) program. Am J Med Genet B Neuropsychiatr Genet.

[71] Kim JW (2007). Investigation of parent-of-origin effects in ADHD candidate genes. Am J Med Genet B Neuropsychiatr Genet.

[72] Schimmelmann BG (2007). No evidence for preferential transmission of common valine allele of the Val66Met polymorphism of the brain-derived neurotrophic factor gene (BDNF) in ADHD. J Neural Transm.

[73] Weinberg CR, Wilcox AJ, Lie RT (1998). A log-linear approach to case-parent-triad data: assessing effects of disease genes that act either directly or through maternal effects and that may be subject to parental imprinting. Am J Hum Genet.

[74] Gjessing HK, Lie RT (2006). Case-parent triads: estimating single- and double-dose effects of fetal and maternal disease gene haplotypes. Ann Hum Genet.

[75] Howey R, Cordell HJ (2012). PREMIM and EMIM: tools for estimation of maternal, imprinting and interaction effects using multinomial modelling. BMC Bioinform.

[76] Schaid DJ (1999). Likelihoods and TDT for the case-parents design. Genet Epidemiol.

[77] Wilcox AJ, Weinberg CR, Lie RT (1998). Distinguishing the effects of maternal and offspring genes through studies of “case-parent triads”. Am J Epidemiol.

[78] Weinberg CR (1999). Allowing for missing parents in genetic studies of case-parent triads. Am J Hum Genet.

[79] Hoggart CJ (2014). Novel approach identifies SNPs in SLC2A10 and KCNK9 with evidence for parent-of-origin effect on body mass index. PLoS Genet.

[80] Lawlor DA (2008). Mendelian randomization:using genes as intruments for making causal inferences in epidemiology. Stat Med.

[81] Zuccolo L (2013). Prenatal alcohol exposure and offspring cognition and school performance. A “Mendelian randomization” natural experiment. Int J Epidem.

[82] Cross-Disorder Group of the Psychiatric Genomics, C. (2013). Identification of risk loci with shared effects on five major psychiatric disorders: a genome-wide analysis. Lancet.

[83] Lehn H (2007). Attention problems and attention-deficit/hyperactivity disorder in discordant and concordant monozygotic twins: evidence of environmental mediators. J Am Acad Child Adolesc Psych.

